# Optimization of Ethanolic Extraction of Phenolic Antioxidants from Lychee and Longan Seeds Using Response Surface Methodology

**DOI:** 10.3390/foods12152827

**Published:** 2023-07-25

**Authors:** Samart Sai-Ut, Passakorn Kingwascharapong, Md. Anisur Rahman Mazumder, Saroat Rawdkuen

**Affiliations:** 1 Department of Food Science, Faculty of Science, Burapha University, Chonburi 20131, Thailand; samarts@go.buu.ac.th; 2 Department of Fishery Products, Faculty of Fisheries, Kasetsart University, Bangkok 10900, Thailand; passakorn.ki@ku.th; 3 Food Science and Technology Program, School of Agro-Industry, Mae Fah Luang University, Chiang Rai 57100, Thailand; 4 Department of Food Technology and Rural Industries, Bangladesh Agricultural University, Mymensingh 2202, Bangladesh; 5 Unit of Innovative Food Packaging and Biomaterials, School of Agro-Industry, Mae Fah Luang University, Chiang Rai 57100, Thailand

**Keywords:** antioxidant activity, extraction yield, extractable phenolic compound, DPPH, ABTS, FRAP solvent concentration

## Abstract

Lychee seeds (LS) and longan seeds (LoS) are excellent sources of phenolic compounds (PCs) with strong antioxidant activity (AOA). The aim of this study was to optimize the extraction conditions regarding extraction yield (EY), extractable phenolic compound (EPC), and AOA from LS and LoS using surface response methodology (RSM). Solvent concentration, extraction temperature, time, and solid to liquid ratio were optimized using RSM. Increasing the solid to solvent ratio from 1:05 to 1:40 (*w*/*v*), increased EY for LoS, however, EY did not change from 1:20 to 1:40 for LS. Solid–liquid ratio 1:20 was chosen for this study. Increasing the quantity of solvent leads to higher EPC and FRAP. The results showed that LoS exhibited higher AOA than LS measured as DPPH, ABTS, and FRAP, respectively. Ethanol concentrations and temperatures significantly (*p <* 0.05) affect EY, EPC, and AOA. The results (*R*^2^ > 0.85) demonstrated a good fit to the suggested models and a strong correlation between the extraction conditions and the phenolic antioxidant responses. The ethanol concentrations of 41 and 53%, temperatures of 51 and 58 °C, and the corresponding times of 139 and 220 min were the optimal conditions that maximized the EY, EPC, and AOA from LS and LoS.

## 1. Introduction

Lychee and longan have been grown commercially in Thailand for more than 100 years, which is concentrated in the upper Northern provinces. These fruits are the most important subtropical fruits among the top fruit crops grown in Thailand, which is currently the biggest producer in the world. Thailand produced 43.92 thousand metric tons of lychees in 2022, with the northern area producing the highest, at 40.8 thousand metric ton. For longan, Thailand produced 1700 thousand metric tons in the year 2022, with the northern area producing the highest, at about 1040 thousand metric tons [1]. There is an increase in plantations with good prospects for exporting these crops. Longan production is also considered to be an economically important fruit for export. With the technology to control flowering and yield, the lychee and longan are planned to grow more in other regions in Thailand. Most fresh lychee and longan are consumed locally. Thai longan is well-known for its quality; there is a demand in international markets. Lychee is currently popular among customers because of its flavorful and eye-catching appearance. However, the majority of the by-products from longans and lychees are the pericarp and seeds, which are wasted [2,3]. Both longan and lychee are consumed fresh as well as processed by drying or preserving in syrup because of their sweet and unique flavor. In any event, seed and peel are thrown away as residue. In addition, the contemporarily generated waste from lychee, including 10 to 20% from seeds and pericarp 15%, depending on the variety, has caused serious issues for the environment and the economic viability of the processing industries [4,5]. During the processing of lychee, 30–40% of the by-product is generated, which is typically thrown away by the processing industry [6]. For the food industries, this massive seed generation during lychee processing is an overwhelming challenge. However, seeds are used to cure a number of ailments in traditional Chinese medicine, such as pathogenic colds, stagnant humor, orchitis, neuralgia, testicular enlargement, hernias, and stomach problems [7,8]. Consequently, lychee seeds (LS) have the potential to be an active component in a variety of pharmaceutical and food preparations.

Longan seed (LoS) includes a variety of nutrients, most notably carbohydrates (75.57%) [9]. In addition, it contains moisture (7.40%), crude fiber (7.89%), ash (1.73%), protein (7.17%), and fat (0.23%) [9]. The LoS also includes a number of phenolic acids, including butanoic acid, caffeic acid, geraniin, isomallotinic acid, chebulagic acid, and flavogallonic acid [10,11]. A few research studies showed that high concentrations of polyphenolic compounds, such as gallic acid, corilagin, and ellagic acid [3,12], as well as ethyl gallate, 1-O-galloyl-d-glucopyranose, brevifolin, methyl brevifolin carboxylate, and 4-O-l-rhamnopyranosyl-ellagic acid [13], were found in the LoS. Starch polysaccharides (40.7%), proteins (4.93%), crude fibers (24.5%), lipids (3.2%), and minerals such as 0.28% Mg and 0.21% Ca are all abundant in LSs [14,15]. A significant amount of tocopherol, or vitamin E (9.4%) as well as unsaturated fatty acids was found in LS oil [16]. All of the essential amino acids were available in the LS, with the exception of two unique amino acids named -methylenecyclopropylglycine (-MCPG) and hypoglycin A (HGA), which restrict the usage of lychee seeds in food due to their hypoglycemic impact on human health [17]. The ethaolic extract of LS contains five major phenolic compounds, including some bioactive compounds: gallic acid, epicatechin-3-gallate, (-)-gallocatechin, (-)-epicatechin, and procyanidin B_2_ along with a few minor phenolics such as protocatechuic aldehyde, 5-O-coumaroyl methyl quinate, protocatechuic acid, and daucosterol [18,19]. The bioactive compounds found in LS act as powerful antioxidants and are liable to a variety of biological processes, making it a viable option for use in food formulations.

In addition to being successful, an extraction technique for bioactives from plants should be safe, affordable, and environmentally beneficial. The method and solvent selection play an important role. A crucial step in separating these bioactive chemicals is extraction. The effectiveness of the extraction can be considerably influenced by a variety of parameters, including solvent composition, extraction duration, temperature, solvent to solid ratio, and extraction pressure [20,21,22]. Polyphenols from LoS have been isolated and extracted using a variety of techniques [23]. Sudjaroen et al. [24] used chromatography techniques to separate polyphenols from methanol extracts of LoS. Zheng et al. [13] separated 95% ethanol extract of LoS powder with various solvent. Li et al. [25] identified more feasible methods to isolate bioactive compounds from LoS using alkaline extraction and acid precipitation methods. To find the ideal extract conditions, Potisate and Pintha [26] investigated the effect of solvent and extraction techniques on antioxidant activity of LS. They divided LS into three types: whole seeds, pulp seeds, and pericarp seeds. Conventional and microwave extraction methods were used with different solvent such as distilled water, ethanol, citric acid, and baking soda. Paliga et al. [27] investigated the effect of pressurized solvent (n-butane) on the extraction yield (EY), chemical composition and antioxidant activity (AOA) of LS extract using 7–100 bar and temperature in the range of 25 °C to 70 °C. Type and concentration of solvent, extraction temperature and time, solid-to-liquid ration, pressure, and pH may strongly affect the extraction process. It is necessary to optimize the extraction process before extraction of phenolic compounds [28]. Solvent extraction techniques are regarded as the simplest and most straightforward way to identify antioxidants in plant components. Although modern extraction technology, such as supercritical fluid extraction, enzyme assisted extraction, ultrasonic assisted extraction, microwave assisted extraction as well as some new extraction solvents such as ionic liquid, low eutectic solvent and glycerol have been used by few researchers. The most important controlling variables are typically solvent polarity, solvent concentration, extraction time, and temperature [29,30]. Soong and Barlow [31] utilized solvent extraction methods to extract phenolic compounds and compare AOA between seeds and edible portion of jackfruit, avocado, longan, mango, and tamarind [31]. Nonetheless, Eberhardt et al. [32] claimed that it would be challenging to develop a universally optimal extraction strategy because of the complex internal matrix and variety of antioxidant chemicals found in natural sources. This study tries to use the simplest and traditional solvent extraction method to optimize the ethanolic extraction of phenolic antioxidants from LS and LoS using response surface methodology (RSM).

In general, empirical or statistical approaches can be used to optimize a process, with the former having restrictions on full optimization. Evaluation of the impacts of various process factors and their interactions on response variables is possible using the response surface methodology (RSM) [33]. The RSM is well known for optimizing extraction process. Additionally, RSM is a superb statistical method for maximizing variables, and when applied correctly, it reveals the ideal circumstances for process optimization. Since interactions between the various elements cannot be identified by taking each one into account separately, it is beneficial for identifying the effect of individual or combination of independent variables on the process. The goal of this study was to use RSM to maximize the phenolic antioxidants found in LS and LoS.

## 2. Materials and Methods

### 2.1. Chemicals

Absolute ethanol was supplied by Merck (Darmstadt, Germany). Fluka (Steinheim, Germany) provided the 2,2-azinobis (3-ethyl-benzothiazoline-6-sulfonic acid) (ABTS), 2,4,6-tripyridyl-s-triazine (TPTZ), and Folin–Ciocalteu phenol reagent. Sigma chemical Co. (St. Louis, MO, USA) supplied the 2,2′-diphenyl-picrylhydrazyl (DPPH) and gallic acid.

### 2.2. Seeds Preparation

The LS and LoS were collected from Fanginterfoods Co., Ltd. Chiang Mai, Thailand. Pericarp tissues were removed from the seeds. The seeds were washed by running. Extra water was removed by air drying until all water droplets were entirely evaporated. The seeds were dried using a tray drier (BP-80, KN Thai TwoOp, Bangkok, Thailand) at 50 °C for 24 h. A hammer mill (Model CMC-20) was used to grind the dry seeds into a fine powder. The pulverized seeds were passed through a sieve with a mesh size of 20 (0.84 mm), followed being kept in a high-density polyethylene zipper (Ziploc^®^, San Diego, CA, USA) and stored at −20 °C until further use [34,35].

### 2.3. Extraction of Phenolic Compounds

The impact of the solid-to-liquid ratio on extraction was determined using six ratios (1:5, 1:10, 1:15, 1:20, 1:30, and 1:40 (*w*/*v*)). The solid–liquid ratio was selected based on previous study of Rawdkuen et al. [35]. The entire extraction volume was made of up to 30 mL of ethanol and distilled water (50:50, *v*/*v*) solution. Ethanol is used to extract phenolic antioxidant in compliance with the standards governing the use of food-grade solvents [34]. The suspensions were stirred at 150 rpm for 4 h at room temperature (28 ± 2 °C). The extraction solution was centrifuged (MPW-352R, MPW. MED Instruments, Warszawa, Poland) for 15 min at 8000 rpm [35] The supernatant was filtered using a Buckner funnel and Whatman No. 4 solvent-resistant filter paper. The ratio that produced the highest EPC and AAO value was selected for RSM.

### 2.4. Response Surface Methodology

An experimental plan based on a three-factor/five-level design known as a rotatable central composite design, which included 17 experimental runs, including three replicates at the center point, was used to optimize the extraction of PCs from the LS and LoS by RSM. The ethanol concentration (*X*_1_; 40–80%, *v*/*v* ethanol/water), extraction temperature (*X*_2_; 40–80 °C), and extraction time (*X*_3_; 60–180 min) were the independent variables [35]. Five levels of independent variables were reported in coded and uncoded forms (Table 1). The regression coefficients were obtained by multiple linear regressions after the experimental data were fitted to a 2nd-order polynomial model (Equation (1)). Using Minitab statistical software, the desirability function approach was used to determine the optimal extraction conditions. The STATISTICA Kernel Release 7.0.61.0 EN (StatSoft Inc., Tulsa, OK, USA) was used to develop the response surface plots.

(1) $$Y=\beta_{0}+\overset{3}{\underset{i=1}{\sum }}\beta_{i}X_{i}+\overset{3}{\underset{i=1}{\sum }}\beta_{ii}X_{i}^{2}+\overset{2}{\underset{i-1}{\sum }}\overset{3}{\underset{j=2}{\sum }}\beta_{ij}X_{i}X_{j}$$

where *X*_1_, *X*_2_, and *X*_3_ are the independent variables that influence the response Y’s; and *β*_0_ = intercept; *β_i_* (i = 1, 2, 3) = linear; *β_ii_* (i = 1, 2, 3) = quadratic, and *β_ij_* (i = 1, 2, 3; j = 2, 3) = cross-product terms, respectively.

### 2.5. Validation of the Model

The prediction RSM equations were used to determine the optimal conditions for extracting the phenolic antioxidants from the LS and LoS based on the ethanol concentration, extraction temperature and time (Table 2). Following the determination of the 2nd-order model prediction and the multifactor analysis of variance, the desire function technique was used to determine the optimal extraction conditions.

### 2.6. Determination of EPC

The EPC was measured by following the modified method of Swain and Hillis [36] using Folin-Ciocalteu technique. A quartz vial containing 50 µL of the extract, 200 µL of deionized water, and 50 µL of the Folin-Ciocalteu reagent was filled, and the contents were then well agitated using a Vortex. After allowing the combination to react for 6 min, 500 µL of a 7% (*w*/*v*) Na_2_CO_3_ solution was added, and it was well mixed. For 90 min, the solution was incubated at room temperature in the dark. A UV spectrophotometer (Bio-chrom/Libra S22, Waterbeach, Cambridge, UK) was used to detect the absorbance at 760 nm, and the data were represented in gallic acid equivalents (GAE; mg/100 g dry weight (DW) using a reference curve for gallic acid (0–200 µg/mL). If the observed absorbance value was higher than the linear range of the standard curve, further dilution would be carried out.

### 2.7. Determination of DPPH Radical Scavenging Activity

The method used to measure DPPH radical scavenging activity was somewhat modified from that published by Brand-Williams et al. [37]. Six hundred (600) µL samples were added with 600 µL of 0.20 mM DPPH in 95% ethanol. After vigorous mixing, the mixture was left to remain at room temperature in the dark for 30 min. A UV spectrophotometer (Biochrom/Libra S22, Waterbeach, Cambridge, UK) was used to measure the absorbance of the resultant solution at 520 nm. The sample blank was made in the same way for each concentration, except that ethanol was used in place of the DPPH solution. The gallic acid standard curve was a logarithm between 2 and 25 µg/mL. Results are presented as mg/100 g DW of gallic acid equivalents (GAE). If the observed DPPH value was higher than the linear range of the standard curve, more dilution would be required.

### 2.8. Determination of Ferric Reducing Antioxidant Powder (FRAP)

The FRAP assay was evaluated according to the modified method of Benzie and Strain [38]. Stock solutions were prepared with 20 mM FeCl_3_.6H_2_O solution, 10 mM TPTZ solution, and 300 mM acetate buffer (pH 3.6). 25 mL of acetate buffer, 2.5 mL of TPTZ solution, and 2.5 mL of FeCl_3_.6H_2_O solution were mixed to prepare a workable solution. The mixture, known as the FRAP solution, was incubated for 30 min at 37 °C in a water bath (Mem-mert, D-91126, Schwabach, Germany). 810 µL of FRAP solution was mixed with 90 µL of sample (concentration range of 0.5 to 10 mg/L) and left at room temperature for 30 min in the dark. The ferrous tripyridyltriazine complex (colored product) was quantified at 595 nm using a UV spectrophotometer (Biochrom/Libra S22, Waterbeach, Cambridge, UK). A blank sample for each concentration was prepared by using distilled water instead of FeCl_3_ in the FRAP solution. Gallic acid concentrations between 10 and 100 µg/mL were used to prepare the standard curve. The activity was measured in terms of mg/100 g DW of gallic acid equivalents (GAE).

### 2.9. Determination of ABTS Antioxidant Activity

2,2′-azino-bis-(3-ethylbenzothiazoline-6-sulfonic) acid (ABTS) radical scavenging activity was measured using modified described by Arnao et al. [39]. The stock solutions were prepared by mixing 2.6 mM potassium persulfate solution and 7.4 mM ABTS solution. The two stock solutions were mixed in equal parts to prepare the working solution, which was then left to react for 12–16 h at room temperature and in complete darkness. The solution was then diluted by mixing 5 mL of ABTS solution with 50 mL of methanol. This resulted in an absorbance of 1.1 ± 0.02 units at 734 nm measured by a UV spectrophotometer (Biochrom/Libra S22, Waterbeach, Cambridge, UK). A new ABTS solution was prepared for each test. 950 µL of ABTS solution was mixed with 50 µL of sample (concentration range of 0.5 to 10 mg/L) and left at room temperature for 120 min in the dark. The same procedure was followed to create a sample blank at each concentration; with the exception that methanol was used in place of the ABTS solution. The absorbance was quantified at 734 nm using a UV spectrophotometer (Biochrom/Libra S22, Waterbeach, Cambridge, UK). A gallic acid standard curve was prepared, with concentrations ranging from 2 to 50 µg/mL. The activity was measured in terms of mg/100 g DW of gallic acid equivalents (GAE).

### 2.10. Statistical Analysis

All of the analysis was performed in triplicate and statistical analysis was carried out by analysis of variance (ANOVA). Duncan’s multiple-range test was used to compare means. SPSS software (SPSS 10.0 for Windows, SPSS Inc., Chicago, IL, USA) was used to conduct the analysis.

## 3. Results and Discussion

### 3.1. Selection of Solid-to-Liquid Ratio

**Figure 1:**
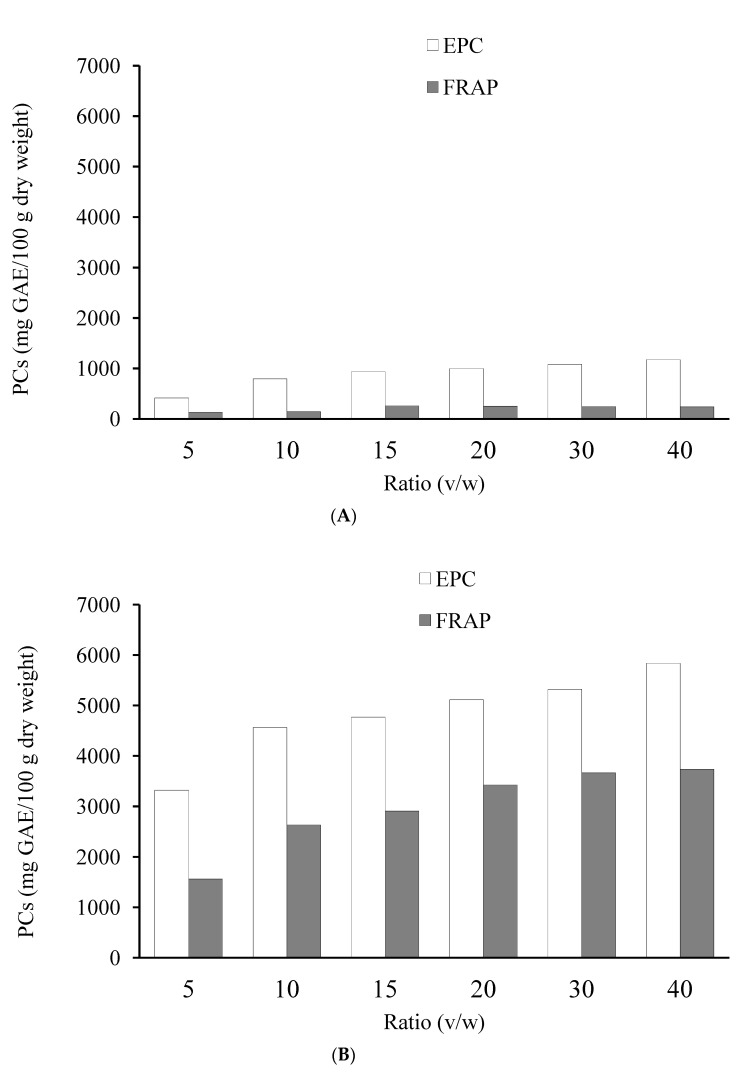
Effect of the solid-to-liquid ratio on the extraction of ferric reducing antioxidant power (FRAP) and extractable phenolic content (EPC) from LS (**A**) and LoS (**B**) using aqueous ethanol (50%, *v*/*v*) at 25 °C for 4 h. GAE = gallic acid equivalents; small letters represents the statistical analysis for EPC and capital letters represent the statistical analysis for FRAP. Bar represents the standard deviation.

Six ratios (1:05, 1:10, 1:15, 1:20, 1:30 and 1:40; *w*/*v*) were tested for their effects on the extraction of PCs from LS and LoS over the course of 4 h at 25 °C and using a 50% (*v*/*v*) ethanol solution as the solvent. The findings demonstrate that the extraction of PCs mainly depends on the solid-to-liquid ratio (Figure 1). Increasing the solid-to-liquid ratio, increased the EPC, and FRAP. This suggests that a higher solid–liquid ratio can increase the solvent–plant material interaction surface area. This allows a higher mass transfer of soluble chemicals from the substance to the solvent. This might also be due to a higher proportion of solvent providing reaction with matrix [40].

The EY from LoS increased significantly (*p* < 0.05) when the amount of solvent was increased (Table 3). The extraction yield from LS did not change significantly (*p* > 0.05) between the ratios of 1:20 to 1:40 (*w*/*v*) (Table 3). Exceeding the solvent concentration of a certain value, leading to the changes polarity of the solvent, resulted in a reduction in the rate of phenolic chemical extraction [41]. The solubility and equilibrium constants of the process are affected by changes in the solid-to-solvent ratio [35]. According to FRAP, the optimal solid–liquid ratio for extracting PCs from LS is between the ratios of 1:20 and 1:30 (*w*/*v*) (Figure 1B). It appears that increasing the quantity of solvent leads to higher EY, EPC, and FRAP antioxidant activity. This might be the reason for the poor solubility of these compounds in the solvent as well as the dependence of isolated compound yields on the predicted dielectric constant of the extraction solvent [42]. The 1:20 solid-to-liquid ratio was hence selected for RSM as a compromise.

### 3.2. Optimization of Extraction of Phenolic Compounds Using RSM

The RSM was used to optimize the extraction of phenolic antioxidants. The obtained results showed that 100 g of dry fruit seeds gave between 5.7 to 8.8 mg EY for LS. This study is supported by a little previous research. For instance, Daorueang [43] showed that EY of LS was 4.6 g/100 g DW. He also found that EPC in the LS was 89.5 mg GAE/g extract and LS inhibited the DPPH radical with an IC_50_ value of 0.65 mg/mL, demonstrating concentration-dependent antiradical action. However, Paliga et al. [27] found that the EY of LS was around 3.5 wt%, total phenolic content of about 126.4 mg GAE/100 g and AOA of up to 78.36%. Similarly, Prasad et al. [44] observed that maximum EY was found at 50% ethanol extract (26.8%), whereas 100% ethanol only produced 23.3%. The EPC of 50% ethanol extract was 239 GAE/g DW. However, there were no significant differences between 50% ethanol and 100% ethanol extract. The percent scavenging activity of 50% ethanol extracts was 48.9%. Therefore, the sequence of the DPPH radical-scavenging activity was 50% ethanol > ethanol > 50% methanol > methanol > BHT > distilled water. It was found that the LS extract exhibited antioxidant power of 134, 581, and 132 GAE/100 g dried seed for DPPH, ABTS and FRAP method, respectively. In general, the main PCs identified in the ethanolic extract of LS epicatechin, gallocatechin, epicatechin-3-gallate, gallic acid, and procyanidin B2 [6].

This study showed that 100 g of dry fruit seeds gave between 8.1 to 15.5 mg EY for LoS. The yield of PCs following traditional solid–liquid extraction was 46.86 mg/g utilizing an alkaline buffer as the extraction solvent; however, acid precipitation showed a phenol separation yield of 22.04 mg/g [25]. Chindaluang and Sriwattana [45] study was on the various extraction techniques for PCs in LoS. The extraction process using ethanol provided the lowest yield (27.7%), which requires the longest extraction time. The yield of the ultrasonic extraction method, which was 35%, was midway between the other two treatments but took the least amount of time, while the yield of the hot water extraction method, which generated the highest LoS extract yield, was 42.8%. While the antioxidant power of LoS extract was found to be 2442, 9173, and 3727 mg GAE/100 g of dried seed for DPPH, ABTS, and FRAP method, respectively. These values were similar with others reporting that LoS contain a phenolic content of 6300 mg GAE/100 g seeds [31].

However, Chindaluang and Sriwattana [45] extracted PCs by ethanol, ultrasonic, and hot water extraction techniques. They found that PCs concentrations were varied with the extraction techniques. For example, PCs for LoS were 11.717, 26.90, and 41.250 mg GAE/mL for ethanol, ultrasonic, and hot water extraction, respectively. In comparison to ascorbic acid (1.37 µg/mL), the AOA of crude extracts of LoS in 20% and 95% ethanol was in the range of 0.82 and 1.73 µg/mL [46]. The major PCs found in the LoS are gallic acid, corilagin, and ellagic acid with retention times of 3.6, 7.8, and 13.2 min, respectively, identified using HPLC by Hong-in et al. [47]. Analysis of variance (ANOVA) for the second order response surface model of each response is shown in Table 4 for both LS and LoS.

It was noted that the response of extraction yield and EPC for LS (EY for LoS) showed significant lack of fit (*p* < 0.05) presenting the model fits not well. However, there were significant (*p* < 0.05) effects on parameters on other responses, e.g., FRAP, DPPH, ABTS. The lack of fit tests, determined by dividing the residual error by the pure error from replicated design points to explain the sufficiency of the relationship between experimental factors and the responses. Table 5 shows the mathematical model for extraction yield; EPC, DPPH, ABTS, and FRAP.

**Figure 2:**
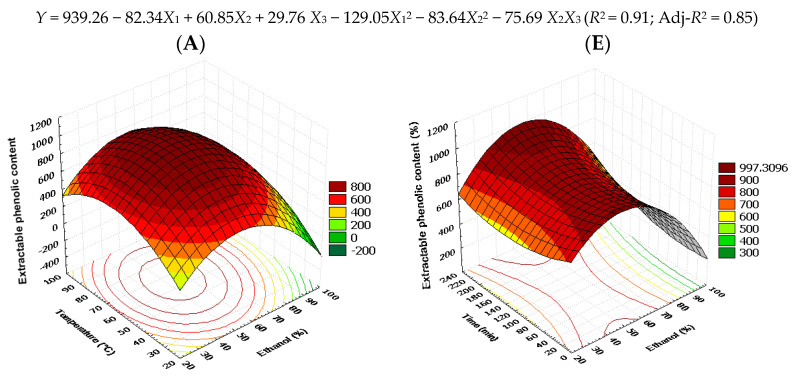

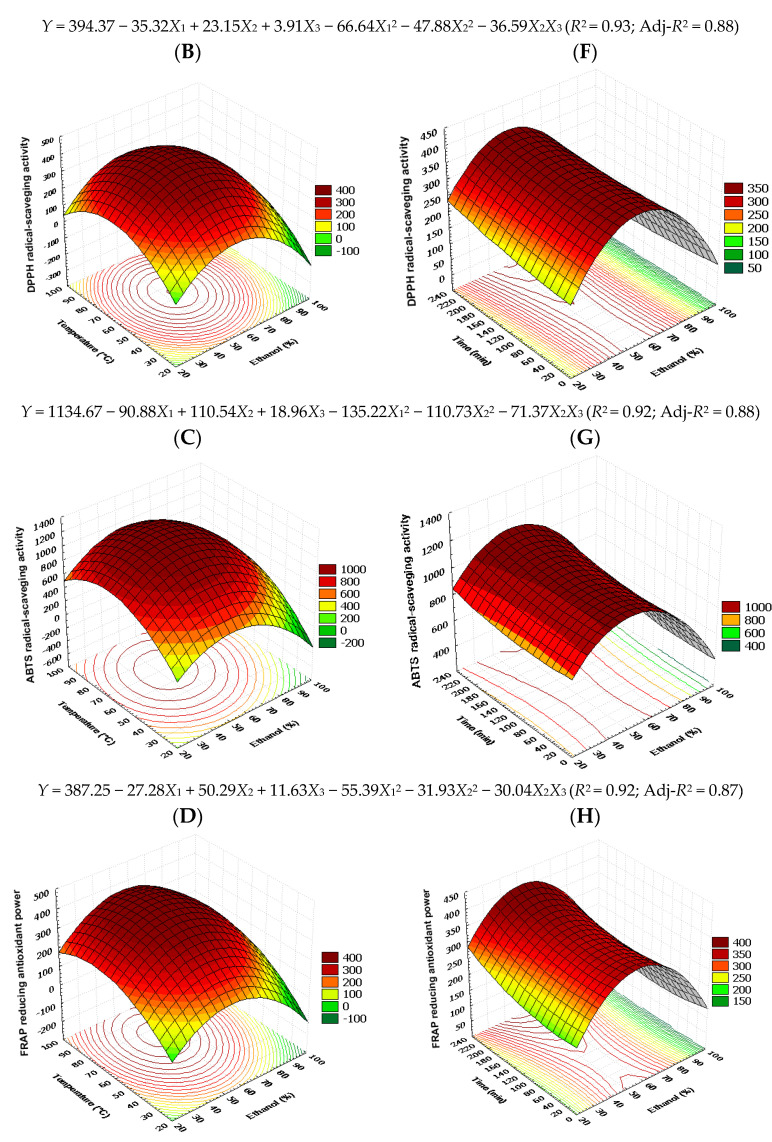
Response surface model plot and regression coefficients of predicted models demonstrating the effects of temperature and ethanol proportion (**A**–**D**) and time and ethanol proportion (**E**–**H**) on EPC, DPPH, ABTS, and FRAP (mg GAE/100 g dry sample) in LS extracts.

**Figure 3:**
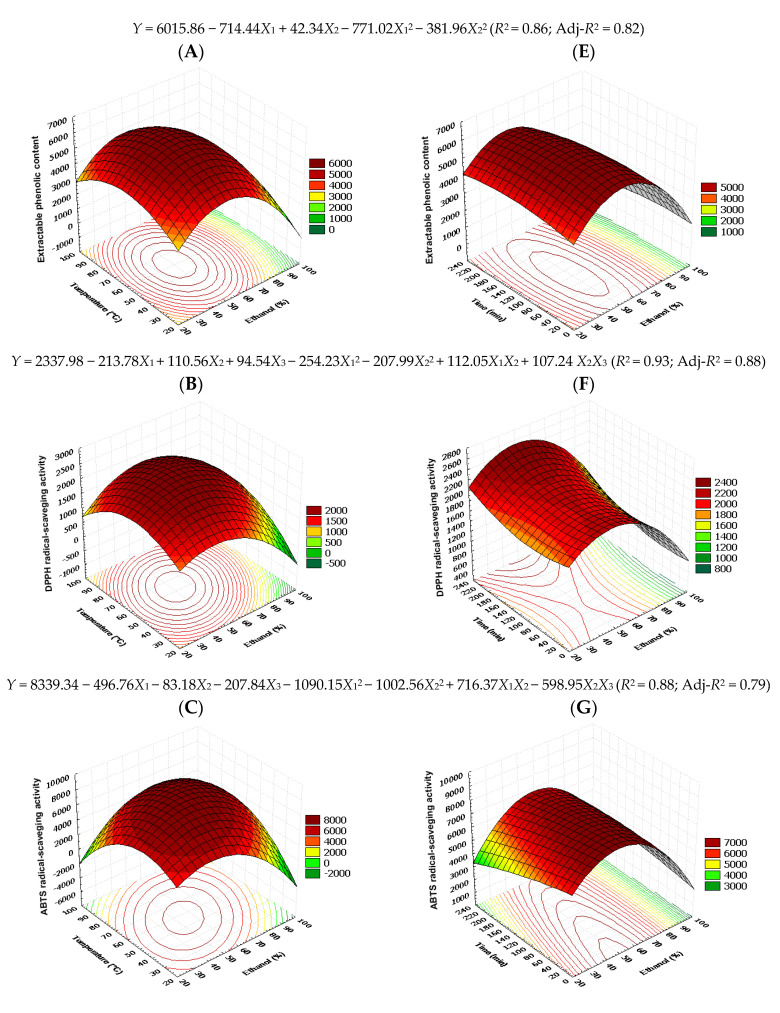

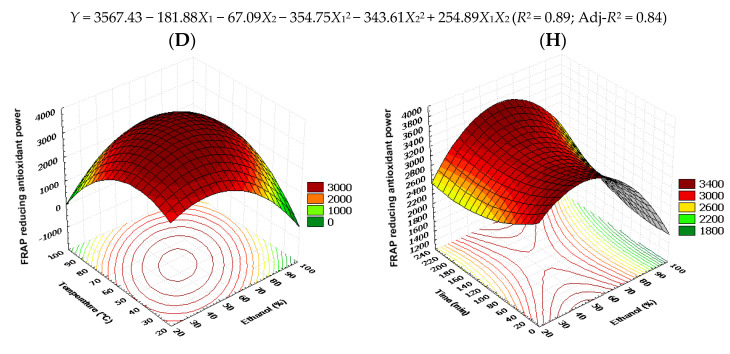
Response surface model plots and regression coefficients of predicted models demonstrating the effects of temperature and ethanol proportion (**A**–**D**) and time and ethanol proportion (**E**–**H**) on EPC, DPPH, ABTS, and FRAP (mg GAE/100 g dry sample) in LoS.

The correlation coefficients (*R*^2^) of each response were higher than 0.85 indicating a good relationship between extraction condition and phenolic antioxidant responses. The data would properly fit with the statistical model if the *R*^2^ values were higher than 0.80 [35,48]. The coefficients of the multiple regression equations that were generated as a result of the independent variables displayed in Table 5. Only the F value for the responses was significant for the model with a confidence level higher than 90 in order for the analysis of variance to fit the quadratic model of the extraction parameters. The adjusted polynomial second order models’ regression coefficients and analysis of variance for antioxidant activity of LS and LoS extracts are summarized in Figure 2 and Figure 3, respectively. According to the surface response analysis for antioxidant activity, temperatures and ethanol concentrations had the greatest impacts (*p* ≤ 0.01 or *p* ≤ 0.001), whereas time had no significant impact (*p* > 0.05). The model’s suitability was confirmed by *R*^2^, Adj-*R*^2^, and a control of model parameters (Table 1).

The relationship between extraction yield and variables is described by the following second order polynomial equation of LS:

Extraction yield (LS) = 7.586 − 0.718*X*_1_ + 0.100*X*_2_ + 0.147*X*_3_ − 0.166*X*_1_^2^ − 0.333*X*_1_*X*_3_ − 0.189*X*_2_*X*_3_. (2)

For the response to DPPH, DPPH, the linear, and quadratic terms of *X*_1_ and *X*_2_ showed statistically significant in coefficients which are described by the following Equation (3):

DPPH (LS) = 394.37 − 35.32*X*_1_ + 23.15*X*_2_ + 3.91*X*_3_ − 66.64*X*_1_^2^ − 47.88*X*_2_^2^ − 36.59*X*_2_*X*_3._ (3)

Similarly, the linear and quadratic for ethanol proportion (*X*_1_) and extraction temperature (*X*_2_) for LoS were the statistically significant coefficients concerning the extraction yield and AAO. Then, the predicted model for extraction yield was calculated by the following Equation (4):

Extraction yield (LoS) = 14.04 − 1.06*X*_1_ + 0.67*X*_2_ − 1.27*X*_1_^2^ − 0.67*X*_2_^2^ − 0.70*X*_1_*X*_2_ (4)

Therefore, the positive linear and quadratic effects of temperature (*X*_2_) suggest that the increase in extraction temperature improved the phenolic compounds yield. Indeed, the solubility and diffusion coefficient of PCs was increased at high temperatures, permitting a higher extraction rate. A positive effect of time (*X*_3_) was detected in LS extract but not significant in that of LoSs. Based on the predicted models, some responses depend on linear terms and quadratic terms, but some depend on interaction terms. It has been documented that ethanol concentrations and temperatures significantly affect the overall antioxidant activities of mango kernels [35], wheat [20], grape cans [21], apple pomaces [22], and wood apples [40].

### 3.3. Effect of Factors on the EPC and AAO

The trend seen for EPC recovery from LS (Figure 2) and LoS (Figure 3) upon simultaneous variation in ethanol and temperature indicates that maximal yield can be achieved at the medium ethanol and temperature values. Most of the PCs available in the plant seeds are phenolic acids, flavonoids, tannins, and polysaccharides, which have a similarity in molecular structure for intermediate polar solvents due to dipole–dipole and dispersion forces, electrostatic, and hydrogen-bonding interactions [49]. At higher ethanol and temperature levels, EPC recovery showed a declining tendency. With regard to the extraction time, maximal EPC for LoS was found for a short duration, while for LS, medium and longer durations were proven favorable, respectively (Figure 3F). Increasing the temperature, increased the amount of EPC and consequently antioxidant activities (*p* < 0.01), although only up to 55 °C. Above this temperature, EPC and AAO reductions were observed. Both seed extracts were primarily impacted by the temperature and ethanol proportion interaction effects. The findings suggested that active chemicals from the solid matrix may be mobilized up to a specific threshold. This could be because of their high-temperature breakdown [20]. Generally, the high temperature enhances the mass transfer rate by decreasing the solvent viscosity. As a result, the solvent distribution to the plant tissues and cell membranes was greater, leading to an increase in phenolic content and antioxidant activities of bioactive compounds [50]. Cacace and-Mazza, 2002. J.E.Cacace and-G.Mazza, Extraction of anthocyanins and other phenolics from black currants with sulfured water, I Agr Food Chem 50 (2002), pp. 5939–5946. Full Text via CrossRef|View Record in Scopus|Cited By in Scopus (33). According to Cacace and Mazza [51], changing the temperature has an impact on a compound’s solubility in the solvent and diffusion coefficient during extraction. As a result, a rise in temperature may enhance the diffusion coefficient and subsequently the rate of diffusion, which would shorten the extraction time [52].

The effects of ethanol proportion, extraction temperature and time on the amount of EPC are shown in Table 4 and Table 5, and Figure 2 and Figure 3. According to Equations (2) and (4), the ethanol concentration showed the highest impression on the extraction of EPC from LS and LoS, as its coefficient has the highest value. Linear and quadratic impacts of the ethanol concentration were seen for the apparent EPC content. The highest EPC was found at a certain ethanol concentration, according to the negative quadratic impact of *X*_1_. In fact, the apparent EPC rose as the ethanol concentration rose, peaked at around 53% ethanol, and then started to fall (Figure 2 and Figure 3). The coefficient of determination for EPC showed a good regression value (*R*^2^ ≥ 0.91 for LS, and 0.86 for LoS). Significant increases in EPC were observed at high ethanol concentrations and low temperatures. To obtain higher EPC, high ethanol concentrations increased the solubility and diffusion of the compounds, which enhanced the solvent ability to extract the compound into the matrix, leading to a decrease at higher temperatures [35]. On the other hand, higher temperatures generally degrade phenolic compounds, leading to a reduction in the EPC (Figure 2A and Figure 3A). The relationships between the EPC and the ethanol content, extraction temperature, and extraction duration are shown in Figure 2 and Figure 3 for LS and LoS, respectively. Strong regression values were shown by *R*^2^ values of 0.91 for LS and 0.86 for LoS in the coefficient of determination for EPC. Low temperatures and high ethanol concentrations significantly increase the EPC. High ethanol concentrations enhance the solubility and diffusion of the chemicals. This enhanced ability of the solvent to extract the molecule into the matrix resulted in a decrease at higher temperatures [36]. However, higher temperatures frequently lead to the degradation of phenolic compounds, which reduced the EPC (Figure 2A and Figure 3A). Furthermore, EPC decreased at extremely high temperatures, most likely as a result of the dwindling dielectric constant, which gave the EPC a less effective contribution [35,53]. Figure 2E and Figure 3E illustrate the effects of time and ethanol concentration on EPC for LS and LoS, respectively. As opposed to this, EPC increases fast when the extraction time is extended from 60 to 139 min for LS and decreases after 140 min. However, it was 60 to 220 min for LoS, before starting to decline after 220 min. This is consistent with the contour plots in Figure 2E and Figure 3E, as well as the findings in Table 5, which show that the interactions between the two variables are not statistically significant (*p* > 0.05). Numerous investigations have found that the amount of ethanol present in the extraction medium affects the yield of EPCs. Research revealed that piceatannol could be extracted most effectively from the seeds of passion fruit using an extraction method that used 80% aqueous ethanol [54]. The effect of the ethanol concentration results from its effect on the polarity of the extraction solvent and the consequent solubility of the phenolic compounds. According to the basic principle of like dissolves like, solvents only extract phytochemicals that are polarly similar to the solvent [55,56]. As with ethanol proportion, temperature also had linear and quadratic effects on the EPC extraction (Table 4 and Table 5). Figure 2 and Figure 3 demonstrate that increase in temperature increased the EPC extraction and peaked at around 51–58 °C, and then started to fall. The rate of diffusion of phenolic compounds may be improved by raising the extraction temperature and by raising the diffusion coefficient [52,57]. Beyond a certain point, however, the temperature may encourage the simultaneous breakdown of PCs that were previously mobilized at a lower temperature or even breakdown of PCs that are still present in the plant matrix [58]. This research shows that EPC only started to degrade when the temperature is above 65 °C (Figure 2 and Figure 3), indicating that it is a somewhat thermo-resistant chemical that can withstand different food preparation procedures. Table 4 and Table 5 show that extraction time had neither linear nor quadratic impact on the EPC extraction. The result suggested that a significant amount of EPC is extracted during the first few minutes of the extraction process. Thus, a study suggests that long extraction periods resulted in lower EPC content. Longer extraction durations may not always result in a complete extraction of phenolic chemicals [59]. Furthermore, phenolic oxidation or degradation brought on by exposure to light, oxygen, or high temperatures may result from lengthy extraction periods [21,58]. DPPH, ABTS, and FRAP were found to obey similar trends such as EPC for LS, but quite different for LoS (Figure 3). The RSM of DPPH (93% for both LS and LoS), ABTS (92% for LS and 88% for LoS), and FRAP (92% for LS and 89% for LoS) data indicated that the model was significant (*p* < 0.01). The two-dimensional contours and three-dimensional representation of the response surfaces of the model are shown in Figure 2 and Figure 3. The DPPH and ABTS assays were almost similar as was shown by the shape of the contour plots. When the ethanol concentration and temperature were raised to 55% and 60 °C, respectively, the DPPH and ABTS assays increased as shown in Figure 2B,F and Figure 3B,F. In general, chemicals may be easily removed from plant cells when the solvent’s polarities are similar to those of the phenolic compounds [60]. Furthermore, a rise in the extractability of bioactive chemicals brought on by a high temperature may improve the antioxidants’ yield [61]. Figure 2F,G and Figure 3F,G illustrate the effects of ethanol concentration and extraction time on the DPPH and ABTS assays. The highest DPPH activities are reached when X_1_ and X_3_ are 55% and 139 min (LS) and 180 min (LoS), respectively, and subsequently the antioxidative activities swiftly increase at first and then decline with increasing ethanol concentration and extraction time. At X_1_ = 65% and X_3_ = 160 min (LS) and 220 min (LoS), the maximum ABTS activities are attained. It goes without saying that excessively high temperatures and ethanol concentrations always reduce antioxidative activity [35]. The response surface plot for the same variables on FRAP shows that the mutual interactions between ethanol concentration (X_1_) and temperature (X_2_) are significant, as seen by the elliptical contour plots in Figure 2D and Figure 3D. Increasing the ethanol concentration and extraction temperature suggests a gradual increase of FRAP assays. As a result, high temperature promotes extraction by increasing the diffusion coefficient and solute’s solubility [62]. Figure 2H and Figure 3H illustrate the linkage between ethanol concentration and extraction time for FRAP assay. With increasing ethanol concentration, an increase in FRAP assay was seen, although the trend slowed after the ethanol concentration reached 65%. Additionally, longer extraction times were seen to result in a higher FRAP value. The effect of temperature and time was quite the opposite, as increases in temperature and time values afforded higher DPPH activity. In particular, DPPH activity from the two seeds was facilitated at medium ethanol proportion and temperature, whereas efficient extraction from LS required an ethanol proportion of around 55%. However, there has been a consistency in ethanol concentration and in all cases; medium ethanol (50%) was demonstrated as the most appropriate. Optimal extraction durations varied from 140 to 220 min (Figure 2 and Figure 3). ABTS values were two to three times higher than DPPH and FRAP values. This might be due to the significant differences in their response to antioxidants, which ABTS and DPPH radicals show similar bi-phase kinetic reactions with many antioxidants, and the FRAP method is based on the reduction of a ferric analogue [63].

### 3.4. Experimental Validation of the Optimal Conditions

The predictive capacities of the model were verified by establishing the optimum condition using the simplex technique and the highest desirability for extraction yield, EPC, DPPH, ABTS, and FRAP antioxidant activity from LS and LoS. The extraction of PCs from LS and LoS has been greatly affected by ethanol concentration, extraction temperature, and extraction time. The optimum condition for ethanolic extraction for LS and LoS were pursued to maximize the EY, EPC, and AOA as determined by the DPPH, ABTS, and FRAP assays. Estimating economic circumstances that will decrease energy and solvent consumption while maintaining higher EY and AOA is plausible given the requirement to lower actual production expenses. The optimal conditions for this specific confluence of variables result in the optimal extraction condition within the target, as produced by the desire function approach. A 95% mean confidence range around the predicted value for those responses encompassed the measured values. Experimental data for the response extraction yield (mg/100 g DW), EPC (mg GAE/100 g DW), DPPH (mg GAE/100 g DW), ABTS (mg GAE/100 g DW) and FRAP (mg/100 g DW) of LS and LoS are shown in Table 6 and Table 7, respectively, under different extraction conditions shown in Table 2. These findings support the capacity of the model to predict the extraction yield, antioxidant activity measured by EPC, DPPH, ABTS, and FRAP from LS and LoS under experimental conditions. The ethanol proportion of 41% and 53%, extraction temperature of 51 °C and 58 °C, and extraction time of 139 min and 220 min, were the optimal conditions that maximized the extraction yields, EPC and AAO from LS and LoS, respectively. For LS and LoS, the equivalent anticipated response values for yield, EPC, DPPH, ABTS, and FRAP under the optimal conditions were 8.9, 14.2; 967, 6144; 383, 2401; 1117, 8353, and 382, 3609, respectively. The consistency of the predicted and experimental results served to verify the RSM model for the extraction process. It was found that the EY found for fruit by-products, including grape seed (19.2%) [64]. At different solvent, temperature and time, total phenolic compounds of the jamun seed were 72 ± 2.5 mg GAE/g seed extract, guarana seed (119 mg GAE/g seed extract), date seed (55 mg GAE/g seed extract), lychee seed (17.9 mg GAE/g seed extract), grape seed (35–65 mg GAE/g seed extract), jackfruit seed (27.7 mg GAE/g seed extract), longan seed (62.6 mg GAE/g seed extract), and tamarind seed (94.5 mg GAE/g seed extract) [46]. The RSM was effectively used to optimize extraction yield and phenolic antioxidant chemicals from LS and LoS. The second order polynomial model well described the experimental data. In terms of effect on extraction performance, the three factors investigated in this study might be prioritized as follows: ethanol concentration > temperature >> extraction time. The EPC was shown to be significantly correlated to DPPH, ABTS, and FRAP.

## 4. Conclusions

The experimental design method was successful in optimizing phenolic antioxidant extraction conditions from LS and LoS. The RSM has been shown to be useful in assessing the influence of three independent variables (ethanol content, extraction temperature, and time). In terms of effect on extraction performance, the three factors evaluated in this study might be prioritized as follows: ethanol concentration > extraction temperature >> extraction time. LS and LoS have high levels of phenolic compounds with antioxidant activity, which may be recovered using solid-to-water extraction. The solid-to-liquid ratio influenced the extraction of phenolic chemicals. A solid-to-liquid ratio of 1:20 (g/mL) had a substantial influence on EY, EPC, and AOA. This was selected as the optimum parameter. The findings of the experiments revealed that ethanol content and temperature had a substantial influence on the response values. The extraction yield of phenolic compounds, EPC, and FRAP rose as the solid-to-liquid ratio increased. The optimized conditions for maximum EY, EPC, and AOA of the LS and LoS were 41% and 53% ethanol concentration, temperatures of 51 °C and 58 °C, and times of 139 and 220 min, respectively. An increase in extraction temperature increased EPC quantity and, as a result, antioxidant activity (*p* < 0.01), but only up to 55 °C and reduced above this threshold. The interaction effects of temperature and ethanol percentage mostly impacted both seed extracts. It is anticipated that optimization techniques may help to extract phenolic antioxidants from LS and LoS more efficiently and hence more cost-effectively. The results suggested that active chemicals from the solid matrix might be mobilized to a certain extent. However, future research should focus on the utilization of novel and green extraction technologies such as supercritical fluid extraction, enzyme assisted extraction, ultrasonic assisted extraction, microwave assisted extraction, and as well as some new extraction solvents such as ionic liquid, low eutectic solvent and glycerol to optimize the extraction of phenolic antioxidant from food matrix.

## Figures and Tables

**Table 1:** Independent variables for optimization with coded and actual values.

Independent Variables	Units	Symbols	Code Levels				
			−α	−1	0	+1	+α
Ethanol concentration	%, *v*/*v*	*X*_1_	26.36	40	60	80	93.64
Temperature	°C	*X*_2_	26.36	40	60	80	93.64
Time	min	*X*_3_	19.09	60	120	180	220.9

**Table 2:** Three-factor, three-level face-centered cube design for response surface methodology.

Standard Order ^a^	Run Order ^b^	*X*_1_	*X*_2_	*X*_3_
		Ethanol Concentration (%)	Temperature (°C)	Time (min)
1	15	40 (−1)	40 (−1)	60 (−1)
2	11	80 (+1)	40 (−1)	60 (−1)
3	4	40 (−1)	80 (+1)	60 (−1)
4	13	80 (+1)	80 (+1)	60.00(−1)
5	1	40 (−1)	40 (−1)	180 (+1)
6	3	80 (+1)	40 (−1)	180 (+1)
7	16	40 (−1)	80 (+1)	180 (+1)
8	6	80 (+1)	80 (+1)	180 (+1)
9	12	26.36 (−α)	60 (0)	120 (0)
10	2	93.64 (+α)	60 (0)	120 (0)
11	14	60 (0)	26.36 (−α)	120 (0)
12	7	60 (0)	93.63 (+α)	120 (0)
13	9	60 (0)	60 (0)	19.09 (−α)
14	5	60 (0)	60 (0)	220.90 (+α)
15	8	60 (0)	60 (0)	120 (0)
16	10	60 (0)	60 (0)	120 (0)
17	17	60 (0)	60 (0)	120 (0)

^a^ No randomized; ^b^ Randomized.

**Table 3:** Effect of solid to liquid ratio on the extraction yield from LS and LoS.

Ratio (*w*/*v*)	LS (mg/100 g Sample)	LoS (mg/100 g Sample)
1:05	6.52 ± 0.04 ^a^	8.76 ± 0.17 ^a^
1:10	9.12 ± 0.13 ^b^	11.04 ± 0.13 ^b^
1:15	9.34 ± 0.13 ^b^	11.94 ± 0.07 ^c^
1:20	9.77 ± 0.06 ^c^	12.39 ± 0.21 ^d^
1:30	9.81 ± 0.06 ^c^	13.30 ± 0.16 ^e^
1:40	9.80 ± 0.16 ^c^	13.87 ± 0.42 ^f^

The values are all mean ± SD. Mean values in the same column with various superscripts differ significantly (*p* < 0.05).

**Table 4:** Analysis of variance (ANOVA) of the second order response model of extraction yield, EPC, DPPH, ABTS, and FRAP values from LS and LoS.

Responses	Source	DF ^1^ (LS)	DF ^1^ (LoS)	SS ^2^ (LS)	SS ^2^ (LoS)	MS ^3^ (LS)	MS ^3^ (LoS)	F (LS)	F (LoS)	*p*-Value (LS)	*p*-Value (LoS)
Extraction yield	Lack-of-fit	8	3	0.5453	5.326	0.0685	1.7755	37.21	4.23	0.026	0.046
	Pure error	2	8	0.0037	3.355	0.0018	0.4193				
	Total	16	16	9.5443	56.048						
EPC	Lack-of-fit	8	9	44,518	2,049,453	5565	227,717	247.66	1.99	0.004	0.380
	Pure error	2	2	45	229,404	22	114,702				
	Total	16	16	489,630	17,224,657						
DPPH	Lack-of-fit	8	7	8222	147,221	1027.7	21,032	18.78	5.36	0.052	0.166
	Pure error	2	2	109	7850	54.7	3925				
	Total	16	16	112,382	2,352,154						
ABTS	Lack-of-fit	8	7	43,482	4,209,406	5435	601,344	1.28	7.73	0.511	0.119
	Pure error	2	2	8511	155,653	4255	77,826				
	Total	16	16	686,291	37,479,890						
FRAP	Lack-of-fit	8	3	8232	203,411	1029	67,804	3.85	2.41	0.222	0.142
	Pure error	2	8	534	224,684	267	28,086				
	Total	16	16	105,278	3,914,691						

^1^ Degree of freedom, ^2^ Sum of squares, ^3^ Mean of square. LS = lychee seeds; LoS = Longan seeds. EPC = extractable phenolic content; DPPH = 2,2-Diphenyl-1-picryl-hydrazyl-hydrate; FRAP = ferric reducing antioxidant power; ABTS = 2,2′-azino-bis-(3-ethylbenzothiazoline-6-sulfonic) acid.

**Table 5:** Regression coefficients of predicted models and independent effects of factors for the investigated responses of LS and LoS extracts.

Variables ^a^	Yield CF (LS)	Yield CF (LoS)	EPC CF (LS)	EPC CF (LoS)	DPPH CF (LS)	DPPH CF (LoS)	ABTS CF (LS)	ABTS CF (LoS)	FRAP CF (LS)	FRAP CF (LoS)
*β*_0_	7.586 ***^,b^	14.042 ***^,b^	939.26 ***	6015.86 ***	394.37 ***	2337.98 ***	1134.67 ***	8339.34 ***	387.25 ***	3567.43 ***
*β*_1_	−0.718 ***	−1.062 ***	−82.34 **	−714.44 ***	−35.32 **	−213.78 ***	−90.88 ***	−496.76 *	−27.28 ***	−181.88 **
*β*_2_	0.100 ^ns,c^	0.676 *	60.85 *	42.34 ^ns,c^	23.15 *	110.56 *	110.54 ***	−83.18 ^ns^	50.29 ***	−67.09 ^ns^
*β*_3_	0.147 *		29.76 ^ns^		3.91 ^ns^	94.54 *	18.96 ^ns^	−207.84 ^ns^	11.63 ^ns^	
*β*_11_	−0.166 *	−1.270 ***	−129.05 ***	−771.02 ***	−66.64 ***	−254.23 ***	−135.22 ***	−1090.15 ***	−55.39 ***	−354.75 ***
*β*_22_		−0.670 *	−83.64 **	−381.96 **	−47.88 ***	−207.99 ***	−110.73 ***	−1002.56 ***	−31.93 ***	−343.61 ***
*β*_33_										
*β*_12_		0.701 *				112.05 *		716.37 *		254.89 **
*β*_13_	−0.333 **									
*β*_23_	−0.189 *		−75.69 *		−36.59 *	107.24 *	−71.37 *	−598.95 *	−30.04 *	
*R* ^2^	0.94	0.85	0.91	0.86	0.93	0.93	0.92	0.88	0.92	0.89
Adj-*R*^2^	0.91	0.77	0.85	0.82	0.88	0.88	0.88	0.79	0.87	0.84

^a^ Polynomial model $Y=\beta_{0}+\sum_{i=1}^{3}\beta_{i}X_{i}+\sum_{i=1}^{3}\beta_{ii}X_{i}^{2}+\sum_{i-1}^{2}\sum_{j=2}^{3}\beta_{ij}X_{i}X_{j}$ adjusted by backward elimination at the level of 0.1% using the lack-of-fit test; *β*_0_ = constant coefficient; *β_i_* = linear coefficient (main effect); *β_ii_ =* quadratic coefficient; *β_ij_ =* two factors interaction coefficient; ^b,^* significant at *p* ≤ 0.05; ** significant at *p* ≤ 0.01; *** significant at *p* ≤ 0.001; ^c,ns^ *=* not significant (*p* > 0.05). CF = coefficients; LS = lychee seeds; LoS *=* longan seeds; EPC *=* extractable phenolics content; DPPH = 2,2-Diphenyl-1-picryl-hydrazyl-hydrate; FRAP = ferric reducing antioxidant power; ABTS = 2,2′-azino-bis-(3-ethylbenzothiazoline-6-sulfonic) acid.

**Table 6:** Experimental data for the response extraction yield, extractable phenolic compounds, DPPH, ABTS, and FRAP assay of LS under various extraction conditions as shown in Table 2.

St. Order	Response *									
	Extraction Yield, %		EPC		DPPH		ABTS		FRAP	
	Observed	Predicted	Observed	Predicted	Observed	Predicted	Observed	Predicted	Observed	Predicted
5	8.60 ± 0.1	8.62	841 ± 10	837	335 ± 0.7	333	971 ± 36	959	324 ± 2.5	327
10	5.71 ± 0.2	5.91	338 ± 29	434	134 ± 3.9	146	581 ± 33	599	132 ± 8.3	186
6	7.05 ± 0.4	6.69	765 ± 32	699	252 ± 0.9	262	743 ± 63	778	275 ± 3.6	254
3	7.83 ± 0.3	8.03	911 ± 9	923	361 ± 1.3	371	1138 ± 21	1143	387 ± 6.9	386
14	7.57 ± 0.1	7.89	987 ± 78	990	408 ± 3.4	401	1201 ± 35	1167	398 ± 3.0	403
8	6.38 ± 0.1	6.34	717 ± 31	695	218 ± 4.0	235	845 ± 59	856	330 ± 2.2	305
12	7.60 ± 0.1	7.69	819 ± 24	804	324 ± 2.8	298	1076 ± 11	1007	374 ± 2.7	383
15	7.62 ± 0.1	7.60	940 ± 18	937	406 ± 9.8	394	1097 ± 35	1135	400 ± 6.3	390
13	7.48 ± 0.3	7.39	829 ± 31	902	334 ± 3.8	388	995 ± 31	1103	340 ± 1.1	364
16	7.56 ± 0.2	7.60	938 ± 37	937	420 ± 3.7	394	1168 ± 98	1135	403 ± 7.6	390
2	6.59 ± 0.1	6.69	511 ± 16	463	214 ± 0.9	181	662 ± 93	597	217 ± 2.7	179
9	8.31 ± 0.2	8.33	743 ± 50	706	281 ± 4.6	265	942 ± 38	905	301 ± 20.5	278
4	7.26 ± 0.1	7.09	788 ± 18	737	314 ± 3.4	300	962 ± 38	961	373 ± 8.5	350
11	7.22 ± 0.1	7.36	519 ± 19	611	197 ± 0.7	220	585 ± 87	636	193 ± 10.9	213
1	7.39 ± 0.1	7.28	723 ± 51	690	262 ± 1.5	252	810 ± 100	779	231 ± 10.6	235
7	8.88 ± 0.2	8.62	749 ± 15	792	275 ± 0.5	306	926 ± 10	1038	341 ± 6.4	358
17	7.65 ± 0.4	7.60	947 ± 11	937	407 ± 0.7	394	1227 ± 5	1135	373 ± 17.4	390

* EPC, DPPH, ABTS, and FRAP (mg GAE/100 g dry sample).

**Table 7:** Experimental data for the response extraction yield, extractable phenolic compounds, DPPH, ABTS, and FRAP assay of LoS under various extraction conditions as shown in Table 2.

St. Order	Response *									
	Extraction Yield, %		EPC		DPPH		ABTS		FRAP	
	Observed	Predicted	Observed	Predicted	Observed	Predicted	Observed	Predicted	Observed	Predicted
5	12.21 ± 0.2	13.19	5309 ± 137	5472	2052 ± 171	2078	7569 ± 570	7934	3258 ± 88	3369
10	8.09 ± 0.3	8.67	2606 ± 133	2634	1160 ± 62	1259	3864 ± 657	4420	1977 ± 63	2271
6	9.99 ± 0.5	9.66	4192 ± 63	4043	1552 ± 60	1427	5613 ± 341	5508	2799 ± 15	2698
3	12.97 ± 0.3	13.14	5521 ± 118	5683	1925 ± 89	1886	7140 ± 470	6751	2876 ± 22	2868
14	13.75 ± 0.3	14.04	5979 ± 12	5910	2340 ± 9	2497	7511 ± 285	7990	3591 ± 158	3574
8	12.96 ± 0.3	12.42	3457 ± 38	4128	2177 ± 84	2086	6175 ± 924	5576	3032 ± 50	2921
12	13.28 ± 0.2	13.29	5301 ± 210	5007	1760 ± 31	1936	5324 ± 450	5364	2370 ± 39	2495
15	15.49 ± 0.4	14.04	5965 ± 155	6016	2287 ± 17	2338	9173 ± 62	8339	3563 ± 78	3603
13	13.32 ± 0.1	14.04	5286 ± 194	6122	2127 ± 19	2179	7616 ± 904	8689	3303 ± 222	3474
16	14.29 ± 0.2	14.04	6325 ± 165	6016	2382 ± 73	2338	8697 ± 532	8339	3546 ± 144	3603
2	9.23 ± 0.3	9.66	4124 ± 66	4169	1389 ± 27	1452	5070 ± 989	4726	2433 ± 29	2284
9	13.63 ± 0.2	12.24	5206 ± 167	5037	1898 ± 34	1978	6626 ± 141	6091	3021 ± 54	2882
4	12.47 ± 0.2	12.42	4788 ± 105	4254	1798 ± 6	1683	7090 ± 578	7190	3032 ± 41	2811
11	11.84 ± 0.1	11.01	4711 ± 23	4864	1560 ± 7	1564	5662 ± 209	5644	2692 ± 69	2721
1	12.41 ± 0.4	13.19	5835 ± 16	5598	2177 ± 42	2104	7048 ± 25	7152	3358 ± 35	3360
7	12.25 ± 0.5	13.14	5280 ± 159	5556	2442 ± 5	2290	4329 ± 280	5137	2533 ± 125	2573
17	14.05 ± 0.5	14.04	6642 ± 138	6016	2405 ± 11	2338	8682 ± 98	8339	3727 ± 58	3603

* EPC, DPPH, ABTS, and FRAP (mg GAE/100 g dry sample).

## Data Availability

Data are contained within the article.
